# Experimental evidence for sex-specific plasticity in adult brain

**DOI:** 10.1186/s12983-015-0130-0

**Published:** 2015-12-24

**Authors:** Gábor Herczeg, Abigél Gonda, Gergely Balázs, Kristina Noreikiene, Juha Merilä

**Affiliations:** Behavioural Ecology Group, Department of Systematic Zoology and Ecology, Eötvös Loránd University, Pázmány Péter sétány1/C, 1117 Budapest, Hungary; Ecological Genetics Research Unit, Department of Biosciences, FI-00014 University of Helsinki, Helsinki, Finland

**Keywords:** Brain size, *Gasterosteus aculeatus*, Fish, Neural plasticity, Phenotypic plasticity, Sexual dimorphism

## Abstract

**Background:**

Plasticity in brain size and the size of different brain regions during early ontogeny is known from many vertebrate taxa, but less is known about plasticity in the brains of adults. In contrast to mammals and birds, most parts of a fish’s brain continue to undergo neurogenesis throughout adulthood, making lifelong plasticity in brain size possible. We tested whether maturing adult three-spined sticklebacks (*Gasterosteus aculeatus*) reared in a stimulus-poor environment exhibited brain plasticity in response to environmental enrichment, and whether these responses were sex-specific, thus altering the degree of sexual size dimorphism in the brain.

**Results:**

Relative sizes of total brain and *bulbus olfactorius* showed sex-specific responses to treatment: males developed larger brains but smaller *bulbi olfactorii* than females in the enriched treatment. Hence, the degree of sexual size dimorphism (SSD) in relative brain size and the relative size of the *bulbus olfactorius* was found to be environment-dependent. Furthermore, the enriched treatment induced development of smaller *tecta optica* in both sexes.

**Conclusions:**

These results demonstrate that adult fish can alter the size of their brain (or brain regions) in response to environmental stimuli, and these responses can be sex-specific. Hence, the degree of SSD in brain size can be environment-dependent, and our results hint at the possibility of a large plastic component to SSD in stickleback brains. Apart from contributing to our understanding of the processes shaping and explaining variation in brain size and the size of different brain regions in the wild, the results show that provision of structural complexity in captive environments can influence brain development. Assuming that the observed plasticity influences fish behaviour, these findings may also have relevance for fish stocking, both for economical and conservational purposes.

**Electronic supplementary material:**

The online version of this article (doi:10.1186/s12983-015-0130-0) contains supplementary material, which is available to authorized users.

## Background

Intraspecific variation in brain size and the size of different brain regions have recently been the focus of an increasing number of studies [1–4]. Apart from research directed towards exploring genetically-based evolutionary patterns, environmentally-induced plasticity in the brain has also received a great deal of attention (for reviews see [5–7]). For instance, seasonal variation in the size of certain brain regions has been repeatedly demonstrated [8–12]. Furthermore, evidence for experimentally-induced brain plasticity is also widespread. For example, enrichment of the physical environment has had positive effects on brain development on different anatomical levels in rodents [13–16]. Similar patterns have been reported in salmonids [17–19], where adding a single rock to the rearing tank of juvenile *Onchorhynchus mykiss* resulted in significant cerebellum enlargement [18].

In contrast to higher vertebrates with determinate growth, localized and limited adult neurogenesis [20–22] lower vertebrates with indeterminate growth are characterised by neurogenesis that generally persists longer into adulthood. This facilitates lifelong brain growth, and potentially also plasticity in brain size and the size of different brain regions in response to spatial and temporal environmental variability, even in adults [7, 23–26]. For example, Park et al. [27] reported that adult three-spined sticklebacks (*Gasterosteus aculeatus*) collected from the wild appeared to experience a reduction in relative telencephalon size after 30 days in the laboratory. However, we are not aware of any controlled manipulative experiments investigating phenotypic plasticity in the size of the brain or brain regions in response to stimuli experienced first as adults, as opposed to studies investigating responses to stimuli experienced during early (or entire) development [3, 17, 19, 28–30].

The three-spined stickleback provides a suitable model system to study environmentally-induced brain plasticity, and possible sex differences in it. This is because earlier observational studies of this species have indicated the presence of phenotypic plasticity in the adult brain [27]. Moreover, the sexes of this species differ markedly in their sex roles, as only males build nests, defend territories, and take care of eggs and fry [31]. Correspondingly, a high degree of sexual size dimorphism (SSD) in brain size is seen: although females are on average the larger sex, males can have ca. 23 % larger brains than females [30, 32]. This male-biased SSD in brain size – albeit somewhat lower – has also been shown in the closely related nine-spined stickleback (*Pungitius pungitius*; [3]). Furthermore, anadromous sticklebacks make yearly migrations between structurally very simple pelagic winter habitats and more complex benthic-type summer habitats [31]. Hence, they naturally experience seasonal variation in environmental complexity, which has potential to influence their brain size. However, whether this is actually occurs in sticklebacks, and whether the degree of SSD varies seasonally or in response to different environmental conditions is not currently known.

The aim of this study was to investigate whether environmental enrichment that mimics a shift from a pelagic-type habitat to a benthic-type habitat can induce plastic changes in brain size and structure in adult three-spined sticklebacks, and whether these responses were sex-specific. We hypothesised that sticklebacks would develop larger brains in the enriched environment, and that males (which perform nest-building, express territoriality and parental care) would respond more strongly to environmental stimuli than females, especially in the brain areas that are involved in spatial memory and learning. In other words, we expected to see an increase in the degree of sexual dimorphism in brain size in response to experimental treatments. We further expected to see that the sensory areas important for long distance sensing (e.g. *tecta optica*) would be reduced in the enriched environment that mimicked a benthic habitat due to the increased small-scale spatial complexity. To this end, we reared three-spined sticklebacks in empty aquaria from hatching until the first signs of maturity (ca. 5 months), after which half of the fish were exposed to a new enriched environment for a period of 1 month before quantifying variation in brain size and the size of different brain regions.

## Results

**Fig. 1:**
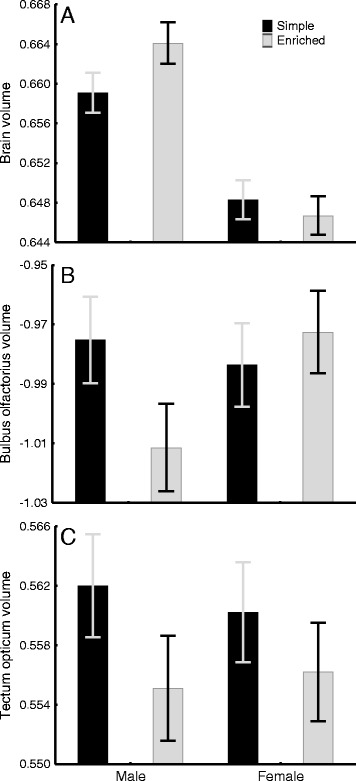
The effects of treatment and sex on stickleback brain development. Least square means (± SE) of **a** Brain (significant sex and sex × treatment effects), **b** *Bulbus olfactorius* (significant sex × treatment effect), **c** *Tectum opticum* (significant treatment effect) volumes are shown

The treatment had a sex-specific effect on total brain volume (Table 1): males had generally larger brains, and only males responded to enrichment by increasing their relative brain size (Fig. 1a). In other words, the male-biased sexual size dimorphism (SSD) in the relative size of the total brain was higher in the enriched (SSD = 4.1 %) than in the simple treatment (SSD = 2.5 %; Fig. 1a). Another sex × treatment interaction was also observed (Table 1): males in the enriched treatment decreased the relative size of their *bulbus olfactorius* compared to the other groups (Fig. 1b). Here, SSD was weakly male-biased in the simple treatment (SSD = 1.9 %) but more pronounced and female-biased in the enriched treatment (SSD = 8.5 %; Fig. 1b). Taken together, significant SSD was only found in the enriched treatment, being either male-biased (total brain) or female-biased (*bulbus olfactorius*), but both patterns were governed by males reacting to the environmental enrichment treatment.

**Table 1:** General linear mixed model (GLMM) results of treatment and sex effects on the stickleback brain

	Total brain	*Bulbus olfactorius*	*Telencephalon*	*Tectum opticum*	*Cerebellum*	*Hypothalamus*
Treatment	1.50 (1214)	1.37 (1217)	0.20 (1223)	**4.19 (1217)**	1.91 (1221)	0.70 (1221)
	0.22 (0.06)	0.24 (0.05)	0.65 (0.01)	**0.042 (0.08)**	0.17 (0.05)	0.40 (0.07)
Sex	**83.34 (1222)**	1.95 (1219)	**34.10 (1225)**	0.02 (1219)	**8.90 (1222)**	**5.13(1222)**
	**<0.001 (0.48)**	0.16 (0.17)	**<0.001 (0.24)**	0.90 (0.02)	**0.003 (0.13)**	**0.024 (0.13)**
Treatment × Sex	**5.56 (1216)**	**5.02 (1215)**	0.42 (1220)	0.28 (1214)	0.08 (1216)	0.29 (1216)
	**0.019 (0.16)**	**0.026 (0.15)**	0.52 (0.04)	0.60 (0.04)	0.77 (0.02)	0.59 (0.04)
Standard length	**293.53 (1223)**	--	--	--	--	--
	**<0.001 (0.75)**					
Total brain	--	**90.24 (1210)**	**303.32 (1143)**	**1021.91 (1213)**	**316.94 (1187)**	**216.24 (1191)**
		**<0.001 (0.55)**	**<0.001 (0.82)**	**<0.001 (0.91)**	**<0.001 (0.79)**	**<0.001 (0.73)**
Marginal *R* ^2^	0.46	0.22	0.51	0.75	0.49	0.43

*F*-statistics with numerator and denominator degrees of freedom in parentheses and *P*-values with effect sizes (*r*) in parentheses are given. Marginal *R* ^2^ is calculated following Nakagawa and Schielzeth [52]. Significant effects are in bold

In both sexes, the enriched treatment decreased the relative size of the visual centre (*tectum opticum*, Fig. 1c; Table 1). Treatment-independent SSD was also found in the relative volumes of the *telencephalon*, *cerebellum*, and *hypothalamus*; males had larger brain regions than females in all cases (Tables 1, 2). However, the degree of SSD was quite low in all of these brain regions (telencephalon = 8.9 %; cerebellum = 4.0 %; hypothalamus = 3.0 %). The degree of male-biased SSD in relative size of the total brain (considering fish in both treatments together) was 3.3 % (Table 2). Our models can be seen as having average explanatory power based on the marginal *R*^2^ values (Table 1), which is expected for traits with high plasticity.

**Table 2:** Sexual dimorphism in the stickleback brain

	Males		Females		SSD
Trait	Mean ± SD; range	corrMean	Mean ± SD; range	corrMean	
*Bulbus olfactorius* (mm^3^)	0.105 ± 0.025; 0.054–0.168	0.101	0.107 ± 0.025; 0.039–0.195	0.105	3.4 %^b^
*Telencephalon* (mm^3^)	1.062 ± 0.184; 0.554–1.579	1.025	0.940 ± 0.167; 0.549–1.573	0.942	8.9 %^a^
*Tectum opticum* (mm^3^)	3.712 ± 0.486; 2.381–5.007	3.619	3.604 ± 0.469; 2.643–5.025	3.616	<0.1 %
*Cerebellum* (mm^3^)	1.017 ± 0.174; 0.626–1.590	0.986	0.944 ± 0.148; 0.654–1.370	0.948	4 %^a^
*Hypothalamus* (mm^3^)	1.287 ± 0.187; 0.806–1.776	1.251	1.207 ± 0.161; 0.825–1.637	1.215	3 %^a^
Total brain (mm^3^)	4.542 ± 0.195; 4.006–4.929	4.588	4.492 ± 0.190; 4.108–4.968	4.441	3.3 %^a,^ ^b^
Standard length (mm)	40.70 ± 2.66; 35.11–46.72	--	42.95 ± 3.07; 36.62–51.82	--	--

Raw means (Mean) ± Standard Deviations (SD) and range are shown first, and corrected means (corrMean) second. Corrected means are back-transformed Least Squares means from the General Linear Mixed Models (GLMMs) ran on lg-transformed variables. Percentage of difference between sexes (SSD) are calculated as [(higher value – lower value) / lower value]*100] using corrected means for brain regions and raw means for standard length. ^a^denotes a significant sex difference, ^b^denotes a significant sex × treatment interaction (see Results). Standard length is added for illustrative purposes, it was a covariate in the GLMMs

## Discussion

The most salient findings of our study were the environmental enrichment-induced brain plasticity in adult sticklebacks, as well as the sex-specificity of some of these effects. As to the sex-specific responses, Jacobs [33] proposed that the sex investing more into reproductive behaviours might be under stronger selection for increased neural capacity. In sticklebacks, males build nests, perform behavioural courtship displays, guard eggs and fry, and maintain a territory. Thus, it is not surprising that males of both three- and nine-spined sticklebacks have larger brains than females [3, 31, 34]. In addition to reproduction-related responses, seasonal-related brain plasticity has been observed for other species in their natural environments [8, 10]. Perhaps the most striking example of this is provided by the song control nuclei in the telencephalon of male canaries (*Serinus canaria*), which almost doubles in size during the spring singing season compared to fall, when these birds do not sing [8]. Hence, our observation that males increased their relative brain size in an environment where territoriality became possible agrees with the previous observations. However, the explanation for the decreased relative size of olfactory bulbs in males exposed to environmental enrichment is not obvious. One possible explanation is that the functions provided by this brain region are not prioritised by males when living in complex environments. Interestingly, plasticity in the relative size of *bulbus olfactorius* was also found in male, but not female nine-spined sticklebacks in response to two feeding treatments (*ad libitum vs*. food-restriction): males decreased their relative *bulbus olfactorius* size when food was provided *ad libitum*, whereas females did not respond to the treatments [3]. Hence, an alternative, but not mutually exclusive explanation for such sex-specific responses can be that the size of female olfactory bulbs are more strongly buffered towards environmental influences, perhaps because olfactory cues are more important for mate choice in female than male sticklebacks [35].

We found that the relative brain size of males exceed that of females, although females are the larger sex. This result agrees with findings from the few studies that have quantified the degree of sexual dimorphism (SSD) in brain size in fish [3, 29, 32, 34, 36]. It is noteworthy that the magnitude of total brain SSD in our study (2.5 – 4.1 %, depending on treatment) was much less than that observed in a study of Icelandic three-spined stickleback populations (23 %; [34]). One possible explanation for these contrasting results is plasticity: there is now ample evidence from many studies – including the present one – that various environmental factors can induce plastic responses in brain size [18, 29, 37], even in a sex-specific manner [3, 30]. The subjects used by Kotrschal et al. [34] where wild-caught and their brains were measured after mating and parental care trials; hence, the high degree of SSD observed in their study could owe to the seasonal increase of the male brain in response to stimuli derived from breeding activities. In contrast, the males in our study were F_1_-generation fish reared in standardised laboratory conditions, and although sexually mature, they had not yet entered into the reproductive cycle (i.e. mating and parental care), which might be required for the SSD in the brain to become fully expressed. The fact that the relative male brain size (and SSD) became increased in response to treatment conditions that mimic the opportunity for breeding supports this interpretation.

Another possible explanation is that the degree of SSD in the three-spined stickleback brain differs considerably between Icelandic and Finnish populations, as it has been shown to differ between wild-caught ‘normal’ and ‘white’ three-spined sticklebacks [32]. However, population differences in relative brain size in sticklebacks have been shown to have a large plastic component [38]. In addition, the only common garden experiment testing for population differentiation in the degree of SSD in sticklebacks found no evidence for it [3]. Taken together, these points suggest that genetically-based differences in the degree of SSD seems an unlikely, or at least poorly supported explanation for the contrasting levels of SSD in this and the earlier studies [32, 34]. The ultimate test of these explanations would be to rear fish from different populations from immature stages through their full reproductive cycle, and quantify the degree of SSD at different phases of their life cycle.

Irrespective of the sex, we observed that the relative size of the *tecta optica* (the main visual centre of the vertebrate brain) was reduced in the enriched environment. This result is not straightforward to interpret. One possibility is that the smaller visual centres are a direct response to the decreased visibility (and shading) caused by the various objects placed in the tanks. This finding parallels the observation that fish larvae reared in darkness respond by developing smaller *optic tectum* [39]. Such responses could be understandable in light of the fact that neural tissue is among the most expensive tissue to develop and maintain [2, 40]. Hence, the relative size of a given brain region should be a good indicator of its importance for fitness in a given ecological context (e.g. [36, 41]) – unnecessarily large brains or brain regions are expected to be strongly selected against. Hence, plastic reduction of visual cortex volume in response to decreased visual demands could be adaptive if it reduces energy expenditure.

Finally, the possibility of trade-offs occurring between different brain regions in response to the enriched treatment is worth consideration: it is conceivable that those areas of the brain that are less needed may decrease in size to accommodate the increase in size of other brain regions that are of greater need in these circumstances. However, while more detailed analyses are beyond the scope this manuscript, the patterns of the observed changes in the relative sizes of different brain regions do not suggest obvious trade-offs. Namely, in spite of the fact that the relative size of male brains increased in response to the enriched treatment, the only significant changes in the relative sizes of various brain regions (*tectum opticum, bublus olfactorius*) were negative. Likewise, although both sexes responded to enrichment by decreasing the relative size of the *tectum opticum*, no corresponding increases in the relative size of other brain regions were observed, and the correlations between the sizes of different brain parts were always positive and relatively high, both in females (average *r* = 0.55) and males (*r* = 0.58).

## Conclusions

The results of this study demonstrate occurence of sex-dependent plasticity in brain of adult three-spined sticklebacks, and that this plasticity influences the degree of sexual dimorphism in brain. The ability of adult fish to change their brain size after an environmental change is also noteworthy from a more practical point of view. First, as already noted earlier [27, 38], brain size variation seen in material collected from the wild should not be used for evolutionary inference because habitat-dependent patterns do not necessary reflect genetically-based adaptations. The results from the current study further strengthen this argument by demonstrating that over a period of only 1 month, exposure to a new environment can impact brain size and the size of brain regions not only in juvenile, but also in adult fish. Second, many fishes are reared in hatcheries either for stock enhancement or conservation purposes. Following development in a stimulus-poor hatchery environment, they cannot cope with challenges faced in the wild due to neural, cognitive and behavioural deficiencies [18, 42–44]. If fish can adaptively change their brain architecture in adulthood, short-term targeted treatments before their release into the wild might enhance their fitness and lead to improved stocking outcomes.

## Methods

### Sampling and breeding

Adult sticklebacks were collected between 7–13 June 2011 from the Baltic Sea (60°11′54″N; 25°08′22″E) and transported to the University of Helsinki. All *in vitro* crosses were made during 14–15 June 2011. Thirty half- and full-sib families (15 sires, 30 dams) were produced. After the fry hatched, two pools were made by mixing five randomly selected fry from every family into each pool (*N* = 150 fry per pool). The pools were housed in separate 2.8 L tanks in an Allentown Zebrafish Rack (Aquaneering Inc., San Diego, CA, USA). On 22 July 2011, the pools were moved to two large plastic tanks (760 × 540 × 400 mm, length, width & height, respectively) equipped with a one-way flow-through water system supplying filtered tap water. To mimic summer conditions and to facilitate growth, fish were kept at 15 °C water temperature and constant light while in the Zebrafish Rack, and under a 20:4 h daily light:dark regime afterwards throughout the whole experiment. Feeding started with live brine shrimp nauplii (*Artemia* sp.), and changed towards an *Artemia*-chopped bloodworm mix and finally, to bloodworms. Food was provided twice a day, *ad libitum* throughout the experiment. The treatments were initiated after ca. 5 months (on 8. November), when the fish approached adult size (>35 mm in standard length, SL; measured from the tip of the mouth to the end of the tail base) and showed the first signs of maturation (bluish colouration for male eyes). Until this point, they developed in tanks devoid of any objects or substrate.

### The experiment

A total of 254 fish (132 and 122 individuals from the two pools) were available for the experiments. Each of the two pools were divided in half and randomly assigned either to a ‘simple’ or ‘enriched’ treatment, resulting in two replicates per treatment. The experimental treatments were carried out in four 317 L aquaria (1400 × 780 × 290 mm in length, width, and height, respectively). In the ‘simple’ treatment, the aquaria were filled with water but left otherwise empty. In the ‘enriched’ treatment, various objects were used to create a complex, stimulus-rich physical environment. The bottoms of the aquaria in the enriched treatment were covered by 30 mm deep gravel substrate. Five 300 mm long grey plastic cylinders (100 mm in diameter with a 50 mm diameter opening on both sides) and five smaller plastic cylinders of various sizes (28 mm in diameter, 90–315 mm in length) were randomly placed vertically and horizontally on the substrate. In addition, four artificial plants, made by attaching 30–40 strips of black plastic (ca. 350 mm long, 20–30 mm wide) to a 50 ml Sarstedt vial filled with sand were placed into aquaria in the enriched treatment. These objects provided not only stimuli, but also decreased visibility by blocking line of sight and increased shading in the aquaria assigned to the enrichment treatment.

### Measurements

One month after being exposed to the treatments (5–9. December), all fish were over-anaesthetised with concentrated (250 mg/L) and sodium bicarbonate buffered MS222 (tricaine methane-sulphonate). The fish were left in the solution for 10 min after cessation of opercular movement, and dissected immediately after this. Their standard length (SL; from the tip of the mouth to the end of the tail base) was measured with a digital calliper to the nearest 0.01 mm. Brains were dissected under a stereomicroscope by removing the top of the neurocranium and severing the cranial nerves and spinal chord. They were fixed in 4 % formalin-0.1 M phosphate-buffered saline solution and measured ca. 2 years later. We were able to obtain brains from 231 individuals (N _simple_ = 114; N _enriched_ = 117). To estimate the brain and the brain region (*bulbus olfactorius*, *telencephalon*, *tectum opticum*, *cerebellum*, *hypothalamus*) volumes, the ellipsoid model [45, 46] based on three-dimensional linear measurements was used as detailed in Noreikiene et al. ([47]; see Additional file 1: Figure S1 for measurement landmarks). Briefly, digital photographs were taken from the dorsal, lateral and ventral sides of the brain from a standard distance and angle, using the same camera and lens. Width, height and length of the brain and the brain regions listed above were measured with TPS.DIG ver1.37 [48]. This data was then fitted to the ellipsoid model [45, 46]. To test the accuracy of our measurements, the process (photography and digital measurements) was repeated three times on 20 randomly chosen fish. Volume estimates were highly repeatable (all R >0.77; *P* < 0.001) indicating high accuracy. The ellipsoid-model-based approach is thought to yield reliable estimates of brain and brain region sizes as verified by comparisons to histology and X-ray micro-computed tomography based estimates [49]. Furthermore, the correlation between brain size estimates based on ellipsoid model estimates and actual brain weights in our data was very high (*r* = 0.94; [47]).

Since individuals from different families were mixed in larger tanks, and the vast majority of the fish at the end of the experiment were not in full breeding condition (and hence, sexing by phenotypic criteria was not reliable), we used microsatellite markers for pedigree reconstruction and sex identification, respectively. The details of these procedures are given in Noreikiene et al. [47]. In short, the individuals were assigned in full-sib families on the basis of allelic variation in seven polymorphic loci using the program Cervus (v. 3.0; [50]). Using the information on possible parental allele combinations, we were able to assign all individuals to their respective families with high confidence (see [47] for details). Sex-identification was based on amplifying a part of the 3′UTR of the NADP-dependent isocitrate dehydrogenase (Idh) locus, which yields two bands for male and one band for female three-spined sticklebacks [47, 51].

### Statistical analyses

All variables were log_10_-transformed before the analyses. General Linear Mixed Models (GLMMs) were used to test for sex and treatment effects. For total brain volume, a model with treatment, sex, their interaction and SL as fixed effects and sire and dam (nested within sire) as random effects was used. For the brain regions, similar models were used, but total brain volume instead of SL was used as a covariate. We also added replicate nested within treatment to the models, but since it was never significant, only results without it are reported. Random effect estimates (for more complex models) are reported in Noreikiene et al. [47], and here we focus on the fixed affects while controlling for non-independence between individuals within families with random effects. In all models, marginal *R*^2^ (i.e. variance explained by the fixed effects; [52]) was used as a goodness-of-fit statistic. To quantify sexual size dimorphism in brain and brain region sizes, percentage of difference [(higher volume – lower volume)/lower volume]*100] based on back transformed least squares mean values provided by the GLMMs were calculated. We note that the experiments did not have any effect on the standard length (GLMM, F_1,13,63_, *P* = 0.34) or body mass (GLMM, F_1,10.3_ = 0.25, *P* = 0.62) of the subjects, and hence, the observed treatment effects (or lack thereof) cannot be explained as being results of simple changes in body size (see also Additional file 2: Figure S2).

### Data accessibility

Primary data underlying this publication is available from Dryad at: doi: 10.5061/dryad.np600.

### Ethical approval

This study was carried out in compliance with international guidelines for experimental research under license (STH223A) from Finnish National Animal Experiment Board.

## Additional files

Additional file 1: Figure S1. A photograph illustring the brain measurements used in estimating volumes of the total brain and different brain regions in three-spined sticklebacks. (DOCX 1337 kb) Additional file 2: Figure S2. Allometric realtionships between (log) total brain volume and (log) standard length (SL) of threespined stickleback females and males in simple (control) and enriched treatments. (DOCX 252 kb)
