# Supplementary material for: Experimental evidence for sex-specific plasticity in adult brain
Source: Front Zool. 2015 Dec 24;12:38. doi: 10.1186/s12983-015-0130-0 (PMC4690261; doi:10.1186/s12983-015-0130-0)

**Additional file 1: Figure S1. A photograph illustring the brain measurements used in estimating volumes of the total brain and different brain regions in three-spined sticklebacks.**

(A) A lateral view illustrating the height (line close to vertical) and length (line close to horizontal) measures of total brain. (B) A lateral view illustrating the height (lines close to vertical) and length (lines close to horizontal) measures of (from left to right, upper regions) *cerebellum*, *tectum opticum*, *telencephalon*, *bulbus olfactorius* and *hypothalamus* (lower region). (C) A dorsal view illustrating width measurment of (from left to right) *cerebellum*, *tectum opticum*, *telencephalon* and *bulbus olfactorius*. (D) A ventral view illustrating the width measure of *hypothalamus*.


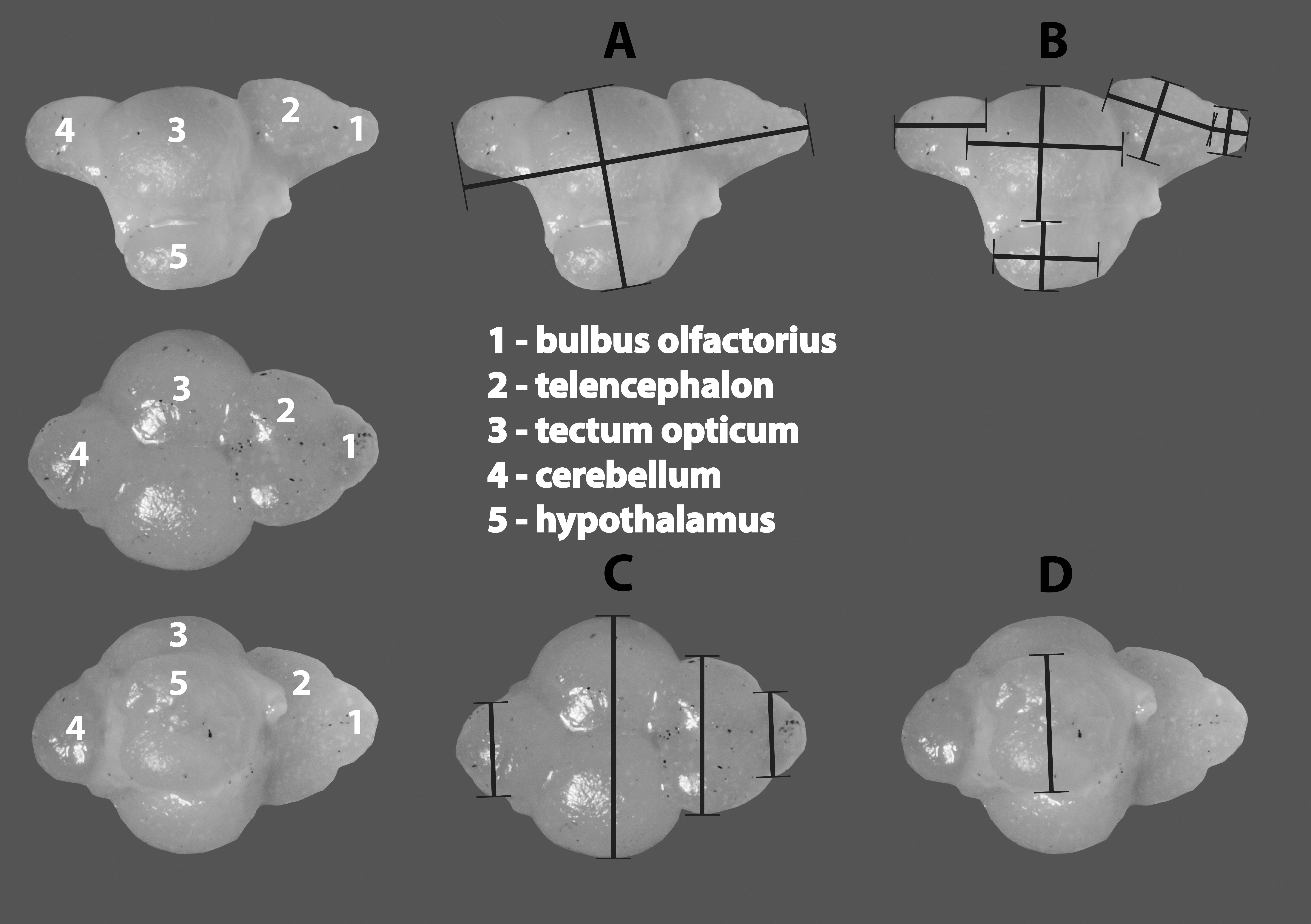

Supplement: Additional file 1: Figure S1. — A photograph illustring the brain measurements used in estimating volumes of the total brain and different brain regions in three-spined sticklebacks. (DOCX 1337 kb) [file 12983_2015_130_MOESM1_ESM.docx]
