# Supplementary material for: Experimental evidence for sex-specific plasticity in adult brain
Source: Front Zool. 2015 Dec 24;12:38. doi: 10.1186/s12983-015-0130-0 (PMC4690261; doi:10.1186/s12983-015-0130-0)
Supplement: Additional file 2: Figure S2. — Allometric realtionships between (log) total brain volume and (log) standard length (SL) of threespined stickleback females and males in simple (control) and enriched treatments. (DOCX 252 kb) [file 12983_2015_130_MOESM2_ESM.docx]

**Additional file 2: Figure S2. Allometric realtionships between (log) total brain volume and (log) standard length (SL) of threespined stickleback females and males in simple (control) and enriched treatments.**


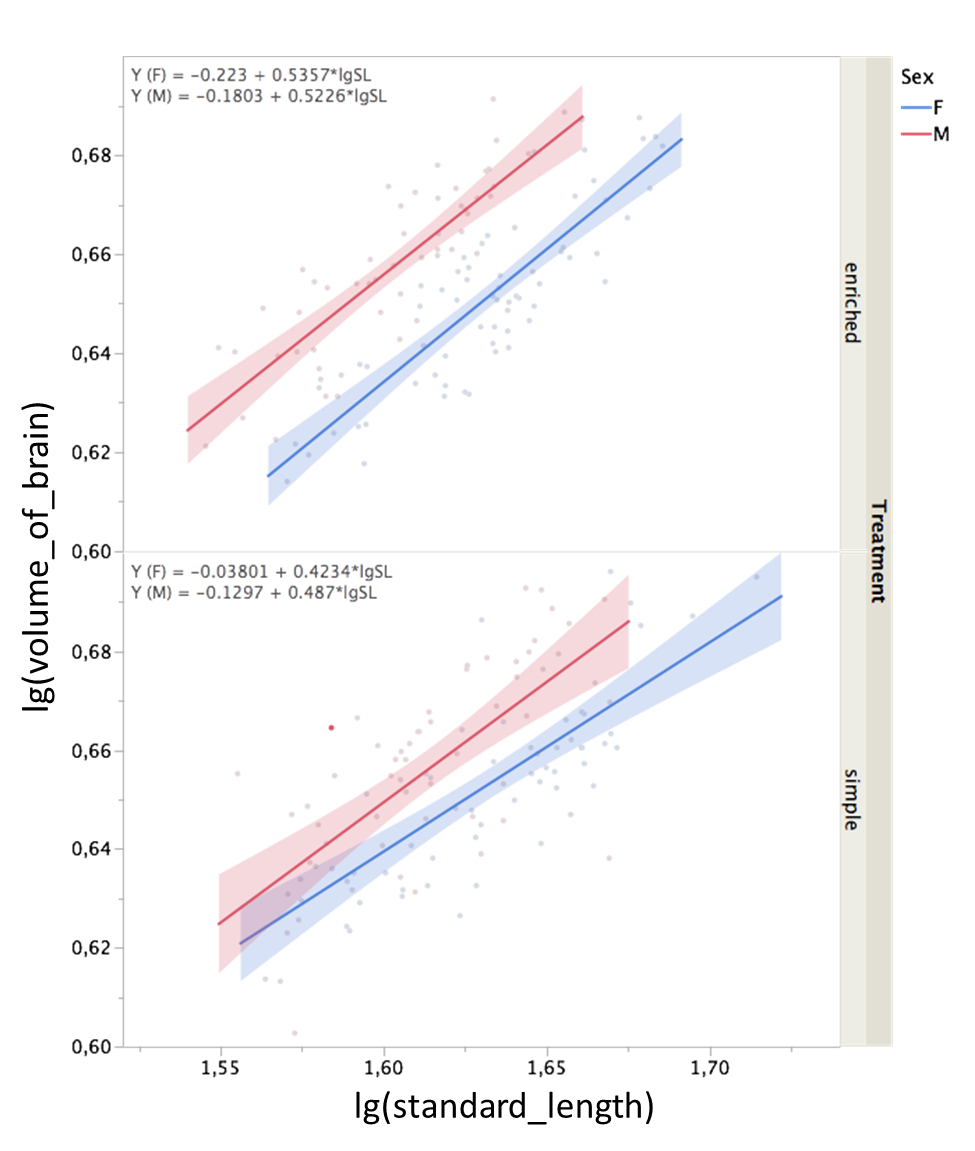
There is no difference in (log) standard length between the treatments (*F*_1,11.63_ = 0.95, *P* = 0.34), but females are signficantly larger than males (*F*_1, 216.1_ = 45.89, *P* < 0.001). Hence, the fact that for a given standard length males show increased brain volume in the enriched treatment relative to that in control (simple) treatment (sex-by-treatment interaction: *F*_1,140.5_= 7.02, *P* < 0.01) must be a consequence of treatment specific effects on male brain, rather than changes in female (relative) brain size. There is no evidence for differences in allometric relationships between (log) brain volume and (log) standard length in respect to sex (sex-by-standard length interaction: *F*_1,200_ = 0.30, *P* = 0.58), treatment (treatment-by-standard length interaction: *F*_1,40.67_ = 2.07, *P* = 0.16) or both (sex-by-treatment-by-standard length interaction: *F*_1,110_ = 0.18, *P* = 0.67). F = female, M = male.
